# Supplementary material for: Prediction of CD44 Structure by Deep Learning-Based Protein Modeling
Source: Biomolecules. 2023 Jun 28;13(7):1047. doi: 10.3390/biom13071047 (PMC10376988; doi:10.3390/biom13071047)
Supplement: Supplementary file 1 [file biomolecules-13-01047-s001.zip › biomolecules-2449584-supplementary.pdf]

**Supplementary Materials**

# Prediction of CD44 Structure by Deep Learning-Based Protein Modeling

**Chiara Camponeschi [1], Benedetta Righino [1], Davide Pirolli [1], Alessandro Semeraro [2], Francesco Ria [3,4] and Maria Cristina De Rosa [1,*]**

[1] Institute of Chemical Sciences and Technologies ''Giulio Natta'' (SCITEC)-CNR, 00168 Rome, Italy;
chiara.camponeschi@scitec.cnr.it (C.C.); benedetta.righino@scitec.cnr.it (B.R.); davide.pirolli@cnr.it (D.P.)

[2] Department of Chemistry and Technology of Drugs, Sapienza University of Rome, 00185 Rome, Italy;
alessandro.semeraro@uniroma1.it

[3] Department of Translational Medicine and Surgery, Section of General Pathology, Università Cattolica del Sacro Cuore, 00168 Rome, Italy; francesco.ria@unicatt.it

[4] Fondazione Policlinico Universitario A. Gemelli IRCCS, 00168 Rome, Italy

* Correspondence: mariacristina.derosa@cnr.it; Tel.: +39-0630155135

**Table S1**. Transcript table for human CD44 (ENSG00000026508)

| | Transcript ID | Name | bp | Protein | CCDS | UniProt |
|---|---|---|---|---|---|---|
| **1** | ENST00000428726.8 | CD44-208 | 5431 | 742aa | CCDS7897 | P16070-1 |
| **2** | ENST00000263398.11 | CD44-201 | 4288 | 361aa | CCDS31457 | P16070-12 |
| **3** | ENST00000415148.6 | CD44-206 | 2369 | 699aa | CCDS31455 | P16070-4 |
| **4** | ENST00000433892.6 | CD44-209 | 2292 | 493aa | CCDS31456 | P16070-10 |
| **5** | ENST00000442151.6 | CD44-211 | 1825 | 294aa | | H0Y5E4 |
| **6** | ENST00000434472.6 | CD44-210 | 1634 | 429aa | CCDS55754 | P16070-11 |
| **7** | ENST00000526025.2 | CD44-222 | 1025 | 78aa | | E9PKC6 |
| **8** | ENST00000352818.8 | CD44-205 | 1023 | 340aa | CCDS55755 | P16070-18 |
| **9** | ENST00000525211.6 | CD44-214 | 874 | 277aa | | H0YCV9 |
| **10** | ENST00000526669.6 | CD44-224 | 870 | 206aa | | H0YD13 |
| **11** | ENST00000279452.10 | CD44-204 | 862 | 287aa | | H0Y2P0 |
| **12** | ENST00000531110.6 | CD44-231 | 806 | 268aa | | H0YEU1 |
| **13** | ENST00000525685.6 | CD44-219 | 729 | 243aa | | H0YEV3 |
| **14** | ENST00000526000.6 | CD44-221 | 721 | 240aa | | H0YDW7 |
| **15** | ENST00000525469.1 | CD44-218 | 705 | 82aa | | H0YE40 |
| **16** | ENST00000278385.10 | CD44-202 | 697 | 232aa | | J3KN83 |
| **17** | ENST00000528455.5 | CD44-228 | 687 | 229aa | | H0YD17 |
| **18** | ENST00000528672.1 | CD44-229 | 586 | 195aa | | H0YCL4 |
| **19** | ENST00000531873.5 | CD44-234 | 584 | 195aa | | H0YD90 |
| **20** | ENST00000533222.5 | CD44-236 | 581 | 193aa | | H0YEA1 |
| **21** | ENST00000526553.6 | CD44-223 | 571 | 191aa | | H0YDV8 |
| **22** | ENST00000525688.5 | CD44-220 | 560 | 186aa | | H0YF08 |
| **23** | ENST00000527889.6 | CD44-226 | 548 | 183aa | | H0YDX6 |
| **24** | ENST00000278386.10 | CD44-203 | 484 | 139aa | CCDS31458 | P16070-19 |
| **25** | ENST00000524922.1 | CD44-212 | 352 | 118aa | | H0YES0 |
| **26** | ENST00000425428.6 | CD44-207 | 1768 | 80aa | | Q86UZ1 |
| **27** | ENST00000528922.1 | CD44-230 | 1319 | No protein | | |
| **28** | ENST00000528086.5 | CD44-227 | 580 | No protein | | |
| **29** | ENST00000525209.5 | CD44-213 | 574 | No protein | | |
| **30** | ENST00000531118.5 | CD44-232 | 877 | No protein | | |
| **31** | ENST00000534296.5 | CD44-238 | 863 | No protein | | |
| **32** | ENST00000527326.1 | CD44-225 | 718 | No protein | | |
| **33** | ENST00000532339.1 | CD44-235 | 589 | No protein | | |
| **34** | ENST00000525293.5 | CD44-216 | 569 | No protein | | |
| **35** | ENST00000525348.1 | CD44-217 | 568 | No protein | | |
| **36** | ENST00000534082.1 | CD44-237 | 564 | No protein | | |
| **37** | ENST00000531141.1 | CD44-233 | 537 | No protein | | |
| **38** | ENST00000525241.1 | CD44-215 | 440 | No protein | | |

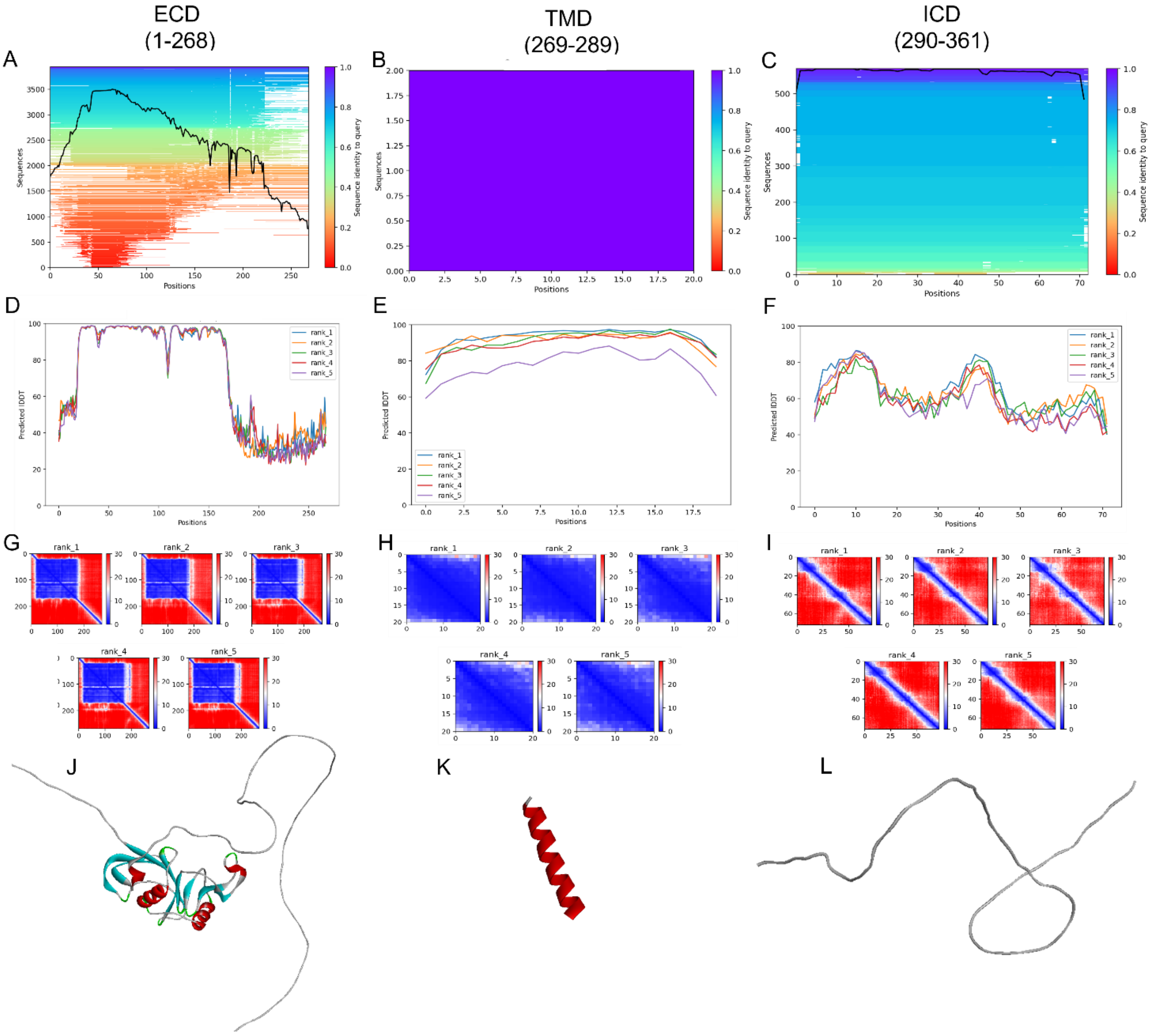


**Figure S1.** AlphaFold2 output for the extracellular (ECD), transmembrane (TMD) and intracellular (ICD) domains of CD44. (A-C) Multiple sequence alignment (MSA). The number of sampled sequences is plotted on the y axis against the amino acid position on the X axis. The sequence identity to the queried sequence is indicated by the bar on the right, colour coded from red (low identity) to blue (high identity). Coverage is shown by the black line. (D-F) Predicted local distance difference test (LDDT) score *vs* position for the five models generated by AlphaFold2. (G-I) Prediction aligned error (PAE) score for the five models generated by AlphaFold2. The axes indicate the position of the amino acids. Reliability of pairwise relative positions of amino acids is color coded from blue (0 Å) to red (30 Å), as shown in the right bar. (J-L) Predicted structural models.

**Table S2.** List of the top 10 threading templates used by D-I-TASSER.

| Rank | PDB Hit | ID1 | ID2 | Cov | Norm. Zscore |
|---|---|---|---|---|---|
| **1** | 1pozA | 1.00 | 0.44 | 12.33 | **1.77** |
| **2** | 1pozA | 1.00 | 0.44 | 12.33 | **2.21** |
| **3** | 5bzfA | 0.86 | 0.35 | 9.98 | **2.22** |
| **4** | 1pozA | 1.00 | 0.44 | 12.33 | 0.52 |
| **5** | 1o7cT | 0.34 | 0.09 | 2.57 | **1.13** |
| **6** | 1o7cT | 0.32 | 0.08 | 2.50 | **1.05** |
| **7** | 2jcqA | 0.87 | 0.36 | 10.06 | **2.17** |
| **8** | 2bkiA | 0.04 | 0.03 | 1.40 | 0.67 |
| **9** | 2pffB | 0.05 | 0.05 | 2.26 | 0.97 |
| **10** | 4p6vF | 0.06 | 0.06 | 2.65 | 0.48 |

ID1 is the number of template residues identical to query divided by number of aligned residues.
ID2 is the number of template residues identical to query divided by query sequence length.
Cov is equal the number of aligned template residues divided by query sequence length.
Norm. Zscore is the normalized Z-score of the threading alignments. A Normalized Z-score >1 means a good alignment and is highlighted in bold.

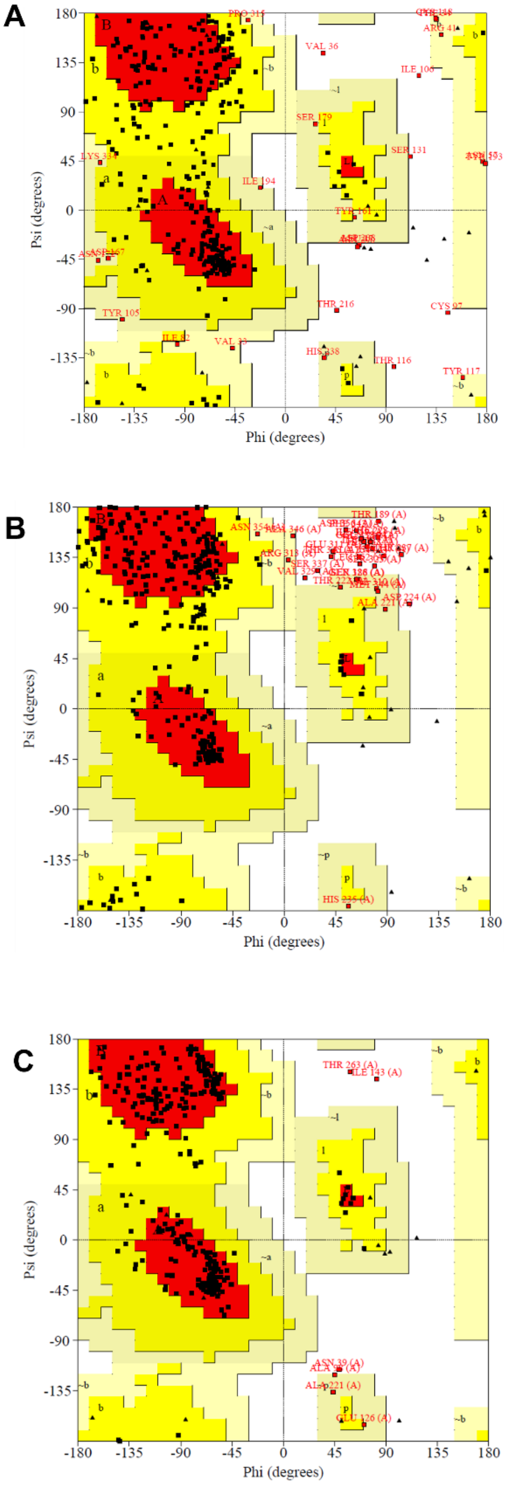


**Figure S2.** Ramachandran plot for the structural models of CD44s generated by (A) D-I-TASSER, (B) AlphaFold2, and (C) RoseTTAFold. The Ramachandran plot shows the phi-psi torsion angles for all residues in the ensemble (except those at the N-terminus). Glycine residues are identified by triangles. Red, dark yellow,, light yellow and white areas indicate the most favourable, allowed,, generously allowed and disallowed, respectively.

**Table S3.** Evaluation of CD44s models by using PROCHECK.

| | Ramachandran Plot statistics (%) | | | |
|---|---|---|---|---|
| | Most favoured | Allowed | generously allowed | disallowed |
| D-I-TASSER | 60.5% | 31.5% | 5.1% | 2.9% |
| AlphaFold2 | 66.9% | 23.8% | 3.2% | 6.1% |
| RoseTTAFold | 87.5% | 10.6% | 1.0% | 1.0% |

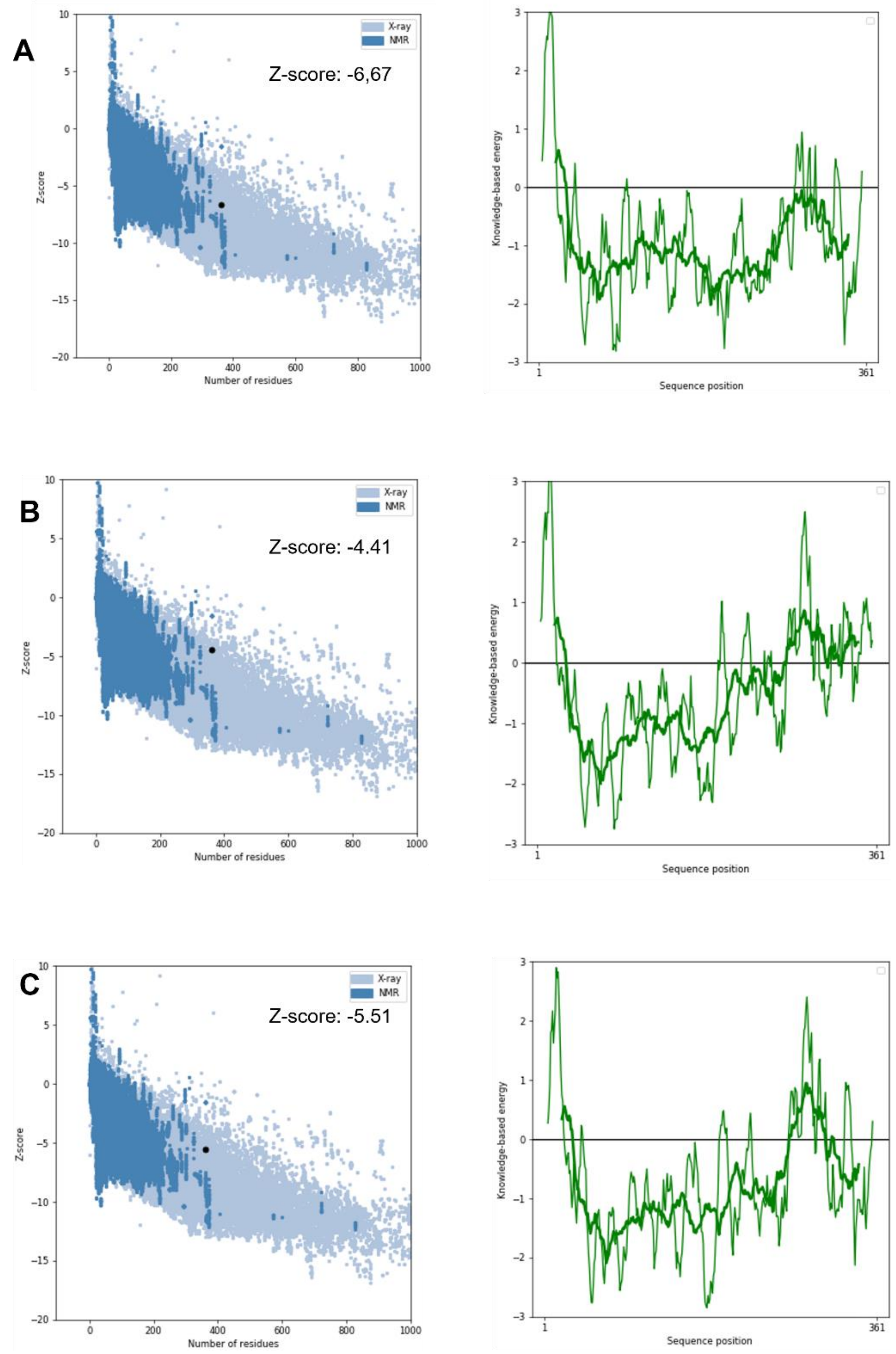


**Figure S3**. Quality assessment of the modeled CD44s using ProSA-Web. Z-scores and energy plots are shown on the left and right side of the panel, respectively, for (A) D-I-TASSER, (B) AlphaFold2 and (C) RoseTTAFold predictions. Z-scores of all protein chains in PDB determined by X-ray crystallography (light) or NMR spectroscopy (dark) with respect to their length, and Z-scores of the modelled structures (large black dots) are highlighted (left). The plot of the energies as a function of amino acid sequence position (thin line) and the residue energies averaged over a sliding window are plotted (thick line) as a function of the central residue in a 40 residue-sized window (right).

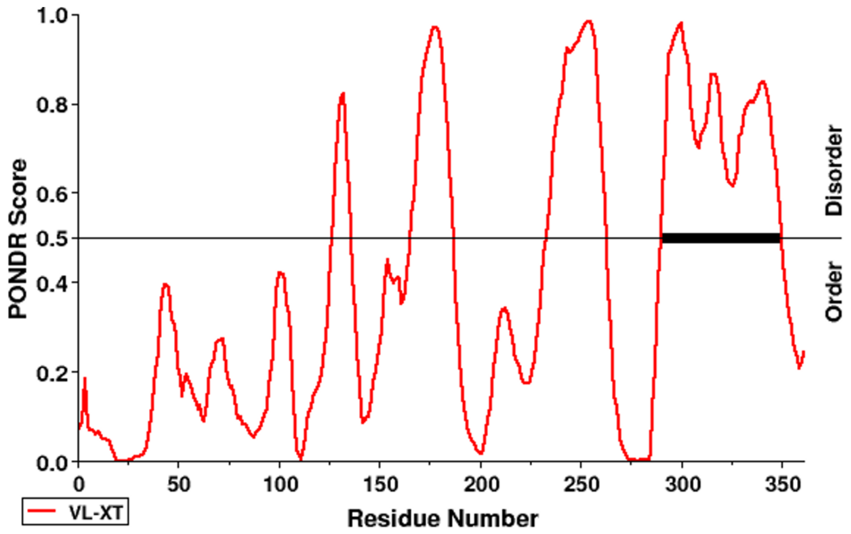


**Figure S4**. Computational prediction of intrinsically disordered regions (IDR) in CD44 with PONDR. A threshold of 0.5 PONDR score establishes the boundary between the ordered and disordered residues. A bold black line indicates predicted IDRs.

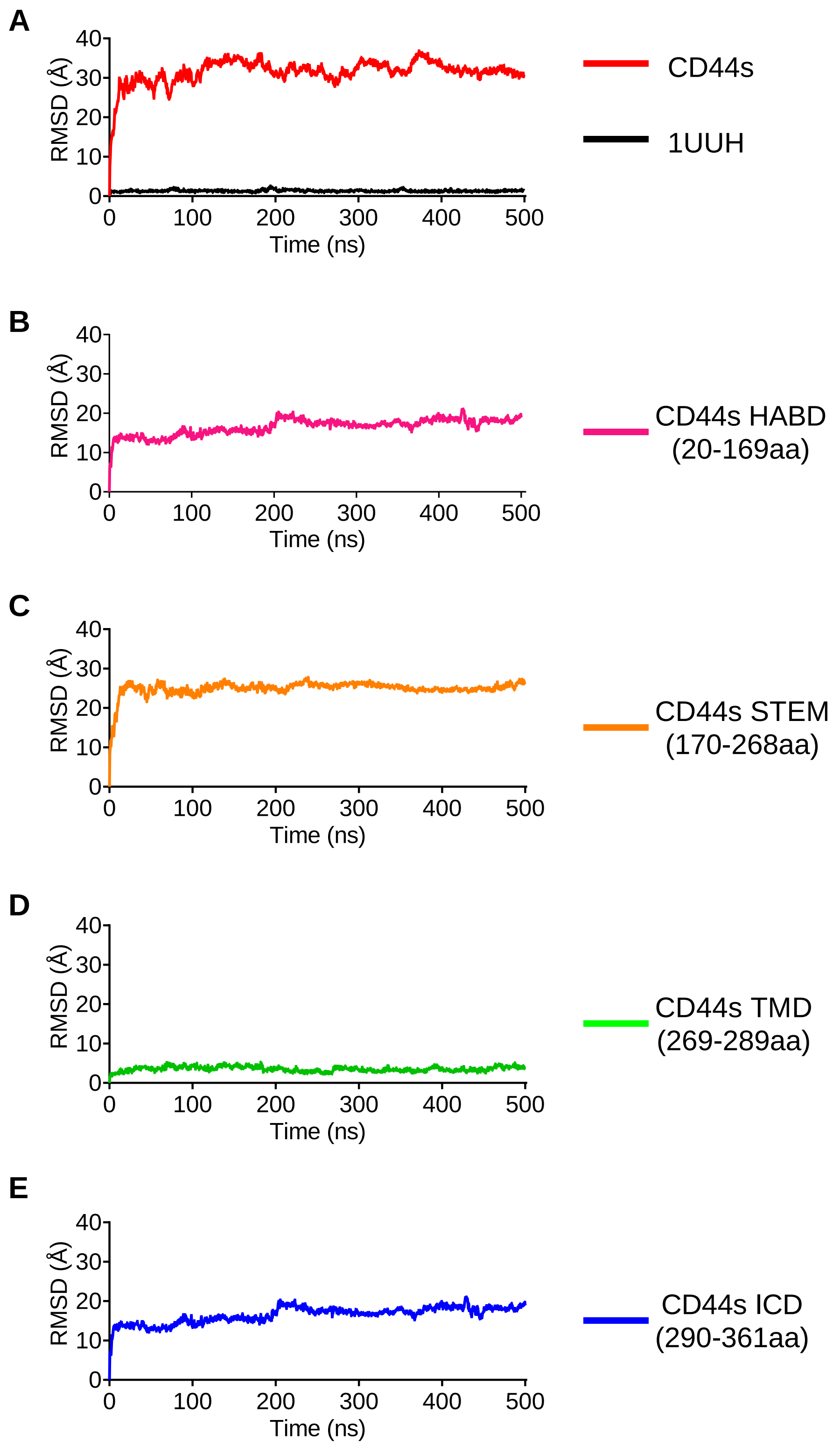


**Figure S5**. Evolution of structural properties over time for MD replicates. C$\alpha$-RMSD of replica 2.

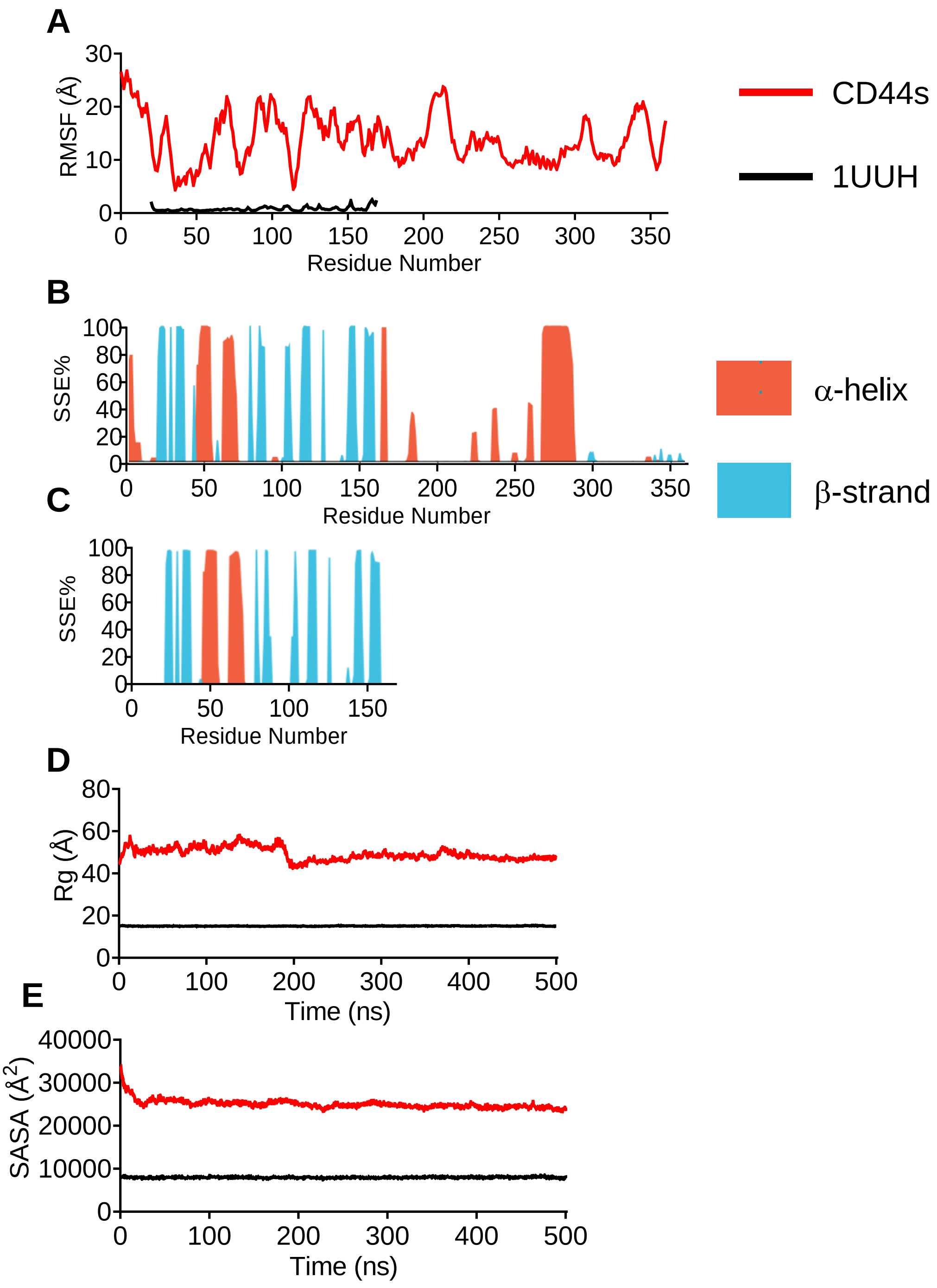


**Figure S6**. Evolution of structural properties over time for MD replicates. Cα-RMSF, SSE, Rg, and SASA of replica 2.

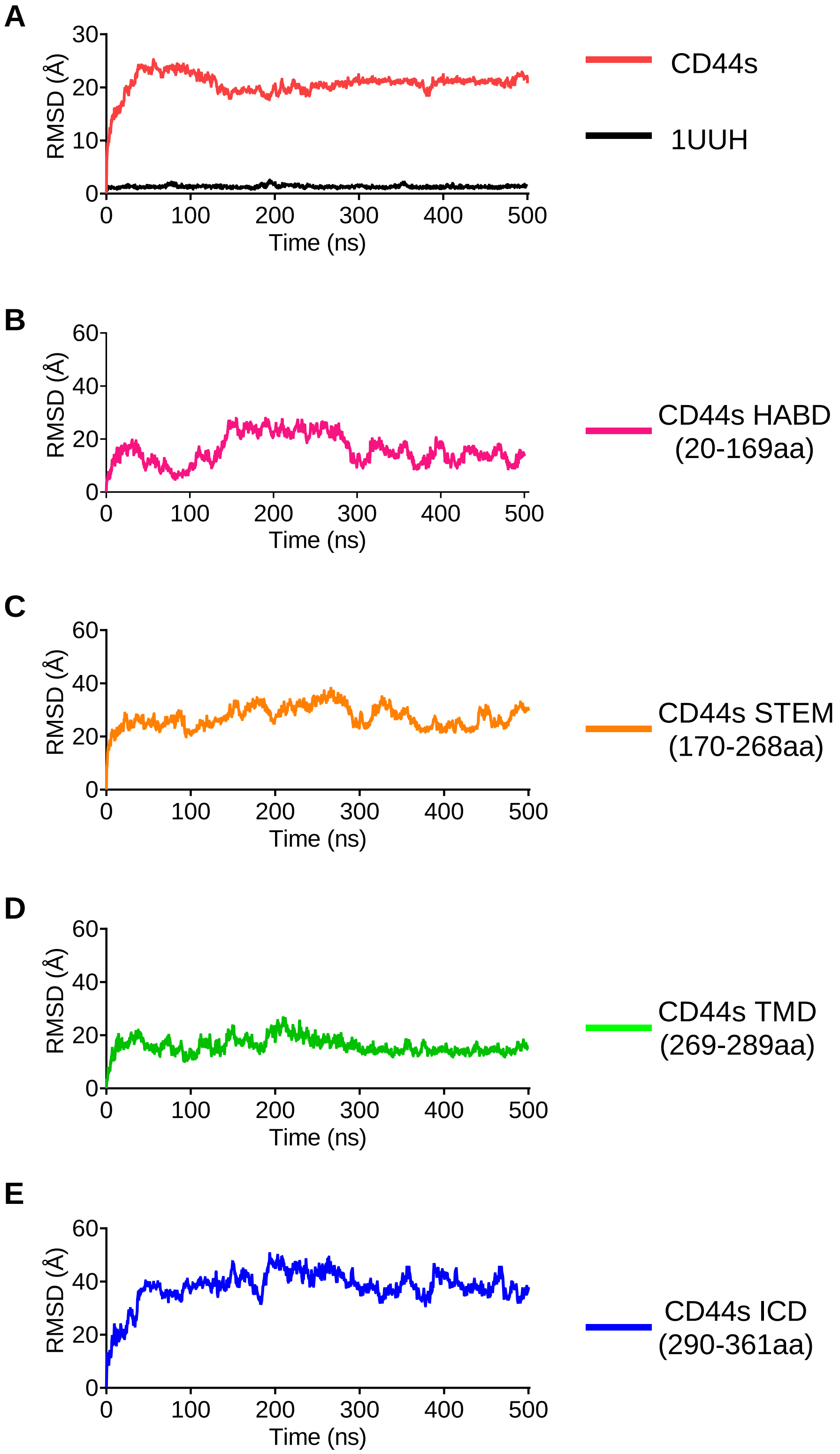


**Figure S7**. Evolution of structural properties over time for MD replicates. C$\alpha$-RMSD of replica 3.

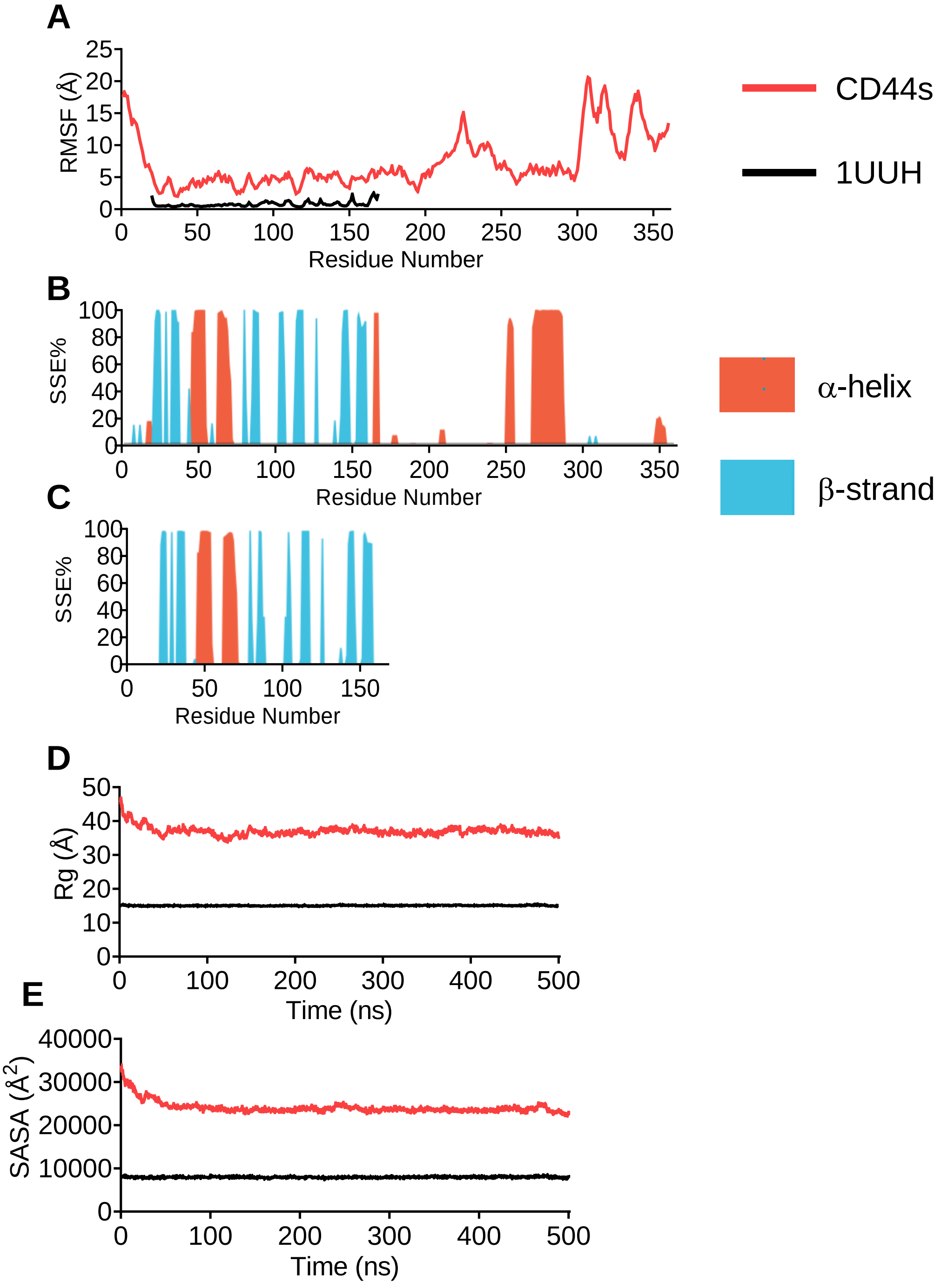


**Figure S8**. Evolution of structural properties over time for MD replicates. Cα-RMSF, SSE, Rg, and SASA of replica 3.
